# Sturge-Weber Syndrome with Osteohypertrophy of Maxilla

**DOI:** 10.1155/2013/964596

**Published:** 2013-05-29

**Authors:** Prashant Babaji, Anju Bansal, Gopal Krishna Choudhury, Rashmita Nayak, Ashok Kodangala Prabhakar, Nagarathna Suratkal, Veena Raju, Suresh S. Kamble

**Affiliations:** ^1^Department of Pedodontics, SPPGIDMS, Lucknow 226001, India; ^2^Department of Pedodontics, Buddha Institute of Dental Sciences, Patna 80001, India; ^3^Department of Prosthodontics, Institute of Dental Sciences, Bhubaneswar 751001, India; ^4^Department of Periodontics, Institute of Dental Sciences, Bubaneswar 751001, India; ^5^Department of Periodontics, Vyas Dental College, Jodhpur 342001, India; ^6^Department of Periodontics, Maharana Pratap Dental College, Gwalior 474001, India; ^7^Department of Oral Medicine, Oxford Dental College & Hospital Bommanahalli, Bangalore 560068, India; ^8^Department of Prosthodontics, MIDSR Dental College, Latur, Maharashtra 413512, India

## Abstract

Sturge-Weber syndrome is a rare nonhereditary developmental condition with neurological and skin disorder, characterized by presence of port wine stain on the face along with ocular disorders, oral manifestations and leptomeningeal angiomas. Here we present an unusual case of Sturge-Weber syndrome with osseous hypertrophy of maxilla.

## 1. Introduction

Sturge-Weber syndrome (SWS) or encephalotrigeminal angiomatosis belongs to group of disorders collectively called as phakomatoses (“mother-spot” disease). This rare congenital neurocutaneous syndrome is characterized by unilateral facial cutaneous vascular malformations affecting the eye and skin in association with ipsilateral leptomeningeal angiomatosis [1, 2]. In 1860, Schirmer first identified this syndrome, and Sturge in 1879 described it in detail; later Frederick Parkes Weber in 1992 demonstrated intracranial calcification [1, 2].

The prevalence is 1 : 50,000 live births. It is equally affected in males and females with no racial predilection [2]. The incidence of osseous involvement in the cutaneous capillary angioma associated with SWS is unknown; however, only few cases have been reported with osseous abnormalities [3–13]. Neoplastic occurrence with vascular malformation is extremely rare but has been reported [3]. Etiology is still unclear [2]. SWS is considered sporadic without genetic abnormalities [3]. It was thought that SWS is caused by persistence of vascular plexus around the cephalic portion of the neural tube, which develops during the sixth week of I.U. life and undergoes regression during the ninth week [1]. Here we report an interesting unusual case of SWS with osseous hypertrophy of maxilla.

## 2. Case Report

**Figure 1:**
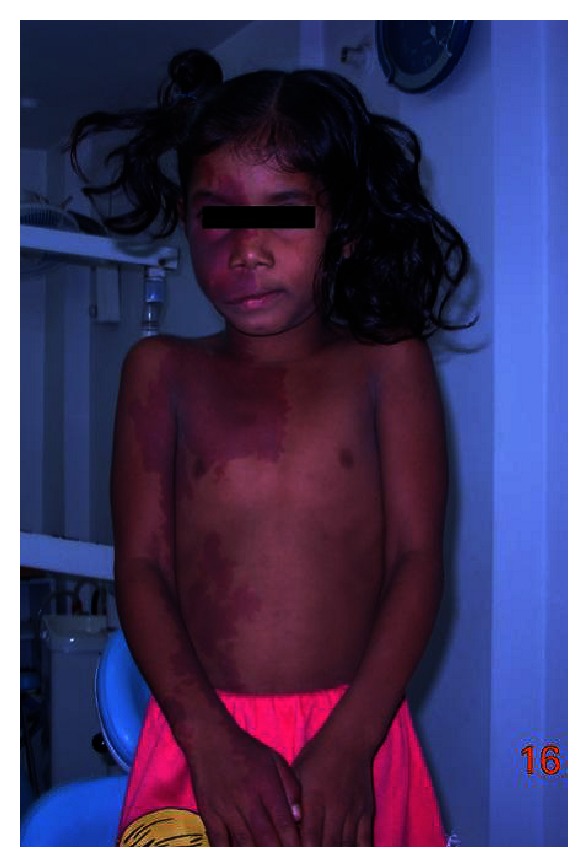
Extraoral unilateral involvement of port wine stain on face, neck, chest, abdomen, and hand.

An 8-year-old female patient reported with osseous abnormalities in the oral cavity. Her history revealed reddish discoloration (port wine stain) on the face since birth and also history of enlarging right maxilla. Medical history revealed that the patient was under medication for convulsion (carbamazepine). There was no visible sign of mental retardation. Family history was noncontributory. Extraoral examination revealed, port wine stain with unilateral (right side) distribution involving forehead, eyelids, cheek, philtrum, upper lip, half of nose, neck, chest, abdomen, and hand. The lower lip and jaw were unaffected (Figure 1). Both eyes appeared normal. Blanching of port wine stains was observed on digital pressure. 

**Figure 2:**
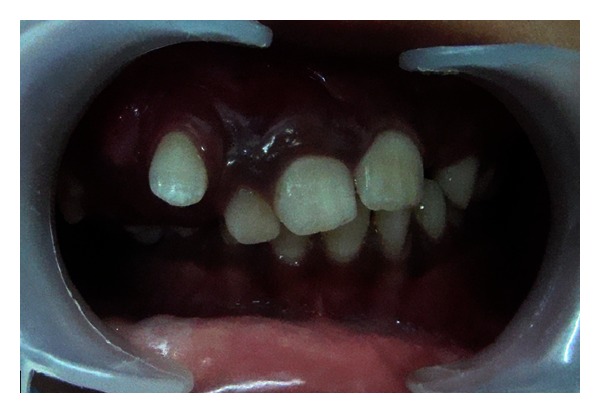
Intraorally unilateral reddish discoloration of gingiva (port wine stain) with osseous enlargement, drifting of teeth, and malocclusion.

**Figure 3:**
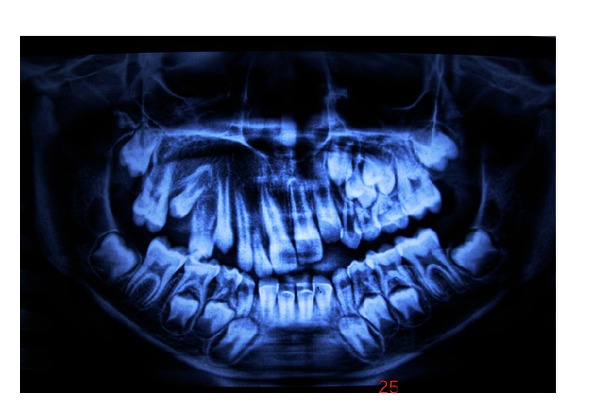
Orthopantamographic view showing the right side osseous enlargement of maxilla with drifting of teeth.

**Figure 4:**
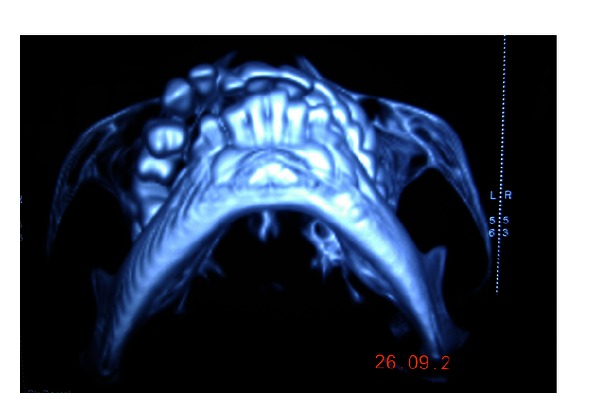
Submandibular view in CT scan showing the right side osseous maxillary alveolar expansion.

Intraoral examination of maxilla on the right side revealed reddish discoloration of gingiva extending from labial frenum to the first molar region with osseous enlargement and drifting of teeth with retained primary upper right central incisor tooth (Figure 2). Gingiva showed overgrowth (hyperplasia) on the right side with bleeding on probing. Gingival enlargement blanched on applying pressure which was suggestive of angiomatous enlargement. Orthopantamographic examination revealed retained upper primary right central incisor, osseous enlargement on the right side of maxilla with drifting of upper permanent right lateral incisor, canine, and first and second premolar teeth (Figure 3). Maxilla showed asymmetric growth with malocclusion. Further CT scan investigation showed facial asymmetry with marked osseous expansion of maxilla (Figure 4). Diagnosis of Sturge-Weber syndrome with osteohypertrophy with gingival hyperplasia was made based on clinical, radiographic, and CT scan investigation.

Maxillectomy was advised for enlarging maxilla. But patient's parents were unwilling for the surgical resectioning of maxilla; hence, the patient was instructed for plaque control measures which included oral prophylaxis at regular interval, oral hygiene instructions, and plaque index scoring. Mobile deciduous right molars were extracted under local anesthesia. Postextraction healing was uneventful.

## 3. Discussion

Sturge-Weber syndrome (OMIM—185300) is an uncommon nonhereditary developmental condition with neurological and skin disorder. It is also known as Sturge-Weber disease, encephalotrigeminal angiomatosis, meningofacial angiomatosis, and Sturge-Weber-Dimitri syndrome [14]. It is a congenital hamartomatous malformation affecting the eye, skin, and central nervous system, with characteristic venous angiomas of leptomeninges, face, jaws, and oral soft tissues. Angiomas of leptomeninges are usually unilateral, located in parietal and occipital region. The presence of angiomas results in alteration of vascular dynamics causing perception of calcium deposition in cerebral cortex underlying the angioma. This can result in the development of seizures, mental retardation, hemiplegia, or hemiparesis [1]. SWS can show “tramline” or gyriform calcifications involving the occipital and parietal lobes on CT, MRI scanning, or on radiographs [2].

Cutaneous angiomas are called as port wine stains, which are having unilateral distribution along dermatomes supplied by the ophthalmic and maxillary division of trigeminal nerve. Sometimes they can be bilateral or can extend up to neck, limb, and other parts of the body as seen in our case [1]. Port wine stains in childhood are classically faint, pink macules, tend to darken to red purple, may be isolated with well-delineated border, or may be very diffuse. Large lesions are warm and may be pulsatile [15]. Port wine stains are named so due to the deep red hue that they leave on skin or mucosa, and such lesions are characterized by profuse bleeding on trauma [1]. Involvement of the area supplied by ophthalmic division is pathognomic and can result in ocular involvement with glaucoma or blindness [1, 14].

Intraorally angiomas can involve lips, buccal mucosa, palate, gingiva, and floor of mouth [1]. Oral changes occur in 40% of SWS cases, involving gingival overgrowth and asymmetric jaw growth [15]. Gingival enlargement might be associated with increased vascular supply. Unilateral hypertrophy of alveolus, pyogenic granuloma, ipsilateral premature eruption or delayed eruption, and malocclusion are the other abnormalities reported [14]. There are very few reported cases with osteohypertrophy as seen in our case with ipsilateral oromaxillofacial osseous overgrowth. Osteohypertrophy is a benign overgrowth of bone. This osteohypertrophy is described as angiodysplasia, and angiodysplastic syndrome, implies a vascular malformation that is associated with secondary changes including further vascular abnormalities and bone hypertrophy which is frequently observed in Klippel-Trenaunay-Weber (KTW) syndrome involving extremities [3].  Table 1 lists various clinical features associated with SWS.

SWS is referred as complete when both CNS and facial angiomas are present and are incomplete when only one area is affected without the other. The Roach scale helps in the classification of the condition [1]. 

*Type I.* Both facial and leptomeningeal angiomas may have glaucoma.

*Type II.* Facial angiomas alone may have glaucoma.

*Type III.* Isolated leptomeningeal angioma usually no glaucoma.

According to the distribution of the vascular malformation, manifestations of SWS were divided into the following four parts: (1) cutaneous manifestations, (2) neurological symptoms and signs, (3) ocular manifestations, (4) other manifestations involving oral cavity [2]. 

The differential diagnosis includes Rendu-Osler-Weber syndrome, angioosteodystrophy syndrome, Maffucci's syndrome, Von Hippel-Lindau disease, Trenaunay-Weber syndrome [1, 14], Bannayan Riley Ruvalcaba syndrome, Divry Van Bogart syndrome and Cobb syndrome [16]. 

Diagnosis is based on imaging studies, CSF analysis for elevated protein, skull radiograph for tram line calcification, cranial CT scan for angioma and calcification. MRI is gold slandered for diagnosis [16].

Treatment and prognosis depend upon severity of clinical condition. Presence of port wine stain can cause psychological trauma to patient. Port wine stains can be treated by dermabrasion, tattooing, and laser therapy [1]. Cryosurgery can be used to correct lip and other soft tissue deformities [14]. Anticonvulsant drugs can be advised for patients with seizures [2]. Aspirin can be advised for headache and to prevent vascular disease [16]. Eye drops are prescribed for glaucoma.

Dental management of the patient should be stressed on behavior management and preventive measures. Poor oral hygiene can lead to secondary inflammatory gingival enlargement and high decayed, missing, and filled teeth (DMFT) score [14]. Gingival overgrowth can be managed by proper oral hygiene maintenance and gingivectomy using Nd:Yag laser [1]. Periodontal injection is preferred in these cases to avoid bleeding. Due to risk of hemorrhage, precautions should be taken during surgical procedures. Absorbable hemostatic agents can be placed at extraction socket [21]; endodontic treatment can be performed since angioma may not involve pulpal tissue; overinstrumentation should be avoided during periapical instrumentation of root canals; and pulpal bleeding can be controlled by cotton pellet and vasoconstrictors [14].

## 4. Conclusion

Management of patients with Sturge-Weber syndrome is challenging due to the risk of hemorrhage. Precautionary measures should be taken to control hemorrhage and complications during surgical procedures. Dental management should include plaque control measure and behavior management.

## Figures and Tables

**Table 1:** Clinical manifestations associated with Sturge-Weber syndrome [1–3, 14–20].

Sl. no	Affected area	Features
1	CNS, cranium	Mental retardation 50%, convulsion 80%, hemiplegia, hemiparesis 30%, leptomeningeal angioma, gyriform calcification (tram line), neuronal loss, cerebral cortex atrophy, cerebral ischemia, and headache
2	Development	Developmental delay
3	Eye	Glaucoma 70%, coloboma of the iris, choroidal hemangioma, buphthalmos, hemianopia, dilated blood vessels, and visual loss
4	Oral cavity	Oral manifestation 40%, port wine stain involving gingiva, buccal mucosa, palate, and floor of mouth and tongue, macroglossia, gingival hyperplasia, bleeding gums, gingival hemangioma, periodontitis, pulpal involvement, osteohypertrophy (rarely), and pyogenic granuloma
5	Skin, face	Unilateral port wine stain involving areas supplied by ophthalmic and maxillary nerves that is, check, lip, and neck.
6	Other extra oral involvement	Port wine stains on neck, chest, abdomen, back, trunk, and extremities
